# Clinical correlation of opposing molecular signatures in head and neck squamous cell carcinoma

**DOI:** 10.1186/s12885-019-6059-5

**Published:** 2019-08-23

**Authors:** Fatima Qadir, Anand Lalli, Huma Habib Dar, Sungjae Hwang, Hebah Aldehlawi, Hong Ma, Haiyan Dai, Ahmad Waseem, Muy-Teck Teh

**Affiliations:** 1 Centre for Oral Immunobiology and Regenerative Medicine, Institute of Dentistry, Barts & The London School of Medicine and Dentistry, Queen Mary University of London, The Blizard Building, 4, Newark Street, London, England E1 2AT UK; 2 China-British Joint Molecular Head and Neck Cancer Research Laboratory, Affiliated Stomatological Hospital of Guizhou Medical University, Guizhou, China; 3 Cancer Research Institute, Affiliated Cancer Hospital and Institute of Guangzhou Medical University, Guangzhou, China

**Keywords:** Molecular diagnostics, Oral squamous cell carcinoma, Tumour heterogeneity, Prognostic biomarkers, Clinical translation, Personalised medicine, Molecular subtypes, Clinical subgroup, Molecular signature, Microarray data mining

## Abstract

**Background:**

The concept of head and neck cancers (HNSCC) having unique molecular signatures is well accepted but relating this to clinical presentation and disease behaviour is essential for patient benefit. Currently the clinical significance of HNSCC molecular subtypes is uncertain therefore personalisation of HNSCC treatment is not yet possible.

**Methods:**

We performed meta-analysis on 8 microarray studies and identified six significantly up- (PLAU, FN1, CDCA5) and down-regulated (CRNN, CLEC3B and DUOX1) genes which were subsequently quantified by RT-qPCR in 100 HNSCC patient margin and core tumour samples.

**Results:**

Retrospective correlation with sociodemographic and clinicopathological patient details identified two subgroups of opposing molecular signature (+q6 and -q6) that correlated to two recognised high-risk HNSCC populations in the UK. The +q6 group were older, male, and excessive alcohol users whilst the –q6 group were younger, female, paan-chewers and predominantly Bangladeshi. Additionally, all patients with tumour recurrence were in the latter subgroup.

**Conclusions:**

We provide the first evidence linking distinct molecular signatures in HNSCC with clinical presentations. Prospective trials are required to determine the correlation between these distinct genotypes and disease progression or treatment response. This is an important step towards the ultimate goal of improving outcomes by utilising personalised molecular-signature-guided treatments for HNSCC patients.

## Background

Head and neck squamous cell carcinoma (HNSCC) is the 6^th^ most common form of cancer worldwide. It is a multifactorial disease, with known risk factors including tobacco, alcohol, areca nut and human papilloma viruses (HPV). As with many cancers HNSCC occurs as a result of abnormal genetic alterations such as point mutations, amplifications, rearrangements and deletions of genes, paving the way for tumour progression [1]. However, no molecular testing technique has yet been developed to aid in early diagnosis and prognostic evaluation of HNSCCs. Whereas, other epithelial origin cancers such as breast and lung already have reliable diagnostic markers (mutant HER2 and EGFR, respectively [2, 3]) which are routinely used by oncologists to personalise treatments and improve outcomes for individual patients. Indeed in the UK, HNSCCs are one of the few cancers where incidence rates are still projected to rise in the future and mortality rates have not decreased despite the significant advances in oncological management (https://www.cancerresearchuk.org/health-professional/cancer-statistics/statistics-by-cancer-type/head-and-neck-cancers).

Efforts are being made to find reliable HNSCC biomarkers that reflect the molecular make-up of the tumours. Through systemic reviews and meta-analysis, EGFR and cyclic D1 have been identified as potential serum diagnostic markers [4], and ANO1 and FADD reported as possible prognostic markers [5]. Genomic changes such as hypermethylation of RAS association domain family protein 1a (RASSF1A), a tumour suppressor gene, has been associated with a high risk of developing HNSCC [6]. However, currently none of these have translated into clinical application.

This study aims to explore the expression of potential HNSCC biomarkers for both diagnostic and prognostic purposes. Meta-analyses of eight independent HNSCC microarray studies was carried out to identify significantly up- and down-regulated genes in studies comparing HNSCC with normal oral mucosa [7–14]. The expression of a panel of likely genes was established in HNSCC patient samples and the molecular findings correlated with each patient’s clinical and histopathological features. We show, for the first time, that within HNSCC patients exist two sub-groups, which are molecularly and clinically distinct from each other. Knowledge of the existence of such heterogeneity will aid in developing personalised treatments to improve outcomes for HNSCC patients.

## Methods

### Clinical samples

The use of fresh clinical specimens collected in the UK was approved by the NHS Research Ethics Committee (06/MRE03/69). All tissue samples were previously collected according to local ethical committee-approved protocols and informed patient consent was obtained from all participants [15, 16]. Fresh tissue biopsies were preserved in RNA*Later* (#AM7022, Ambion, Applied Biosystems, Warrington, UK) and stored short-term at 4 °C (1–7 days) prior to transportation and subsequent storage at − 20 °C until used. All frozen samples were digested with nuclease-free proteinase K at 60 °C prior to mRNA extraction (Dynabeads mRNA Direct kit, Invitrogen).

### Transcriptome data mining

**Fig. 1:**
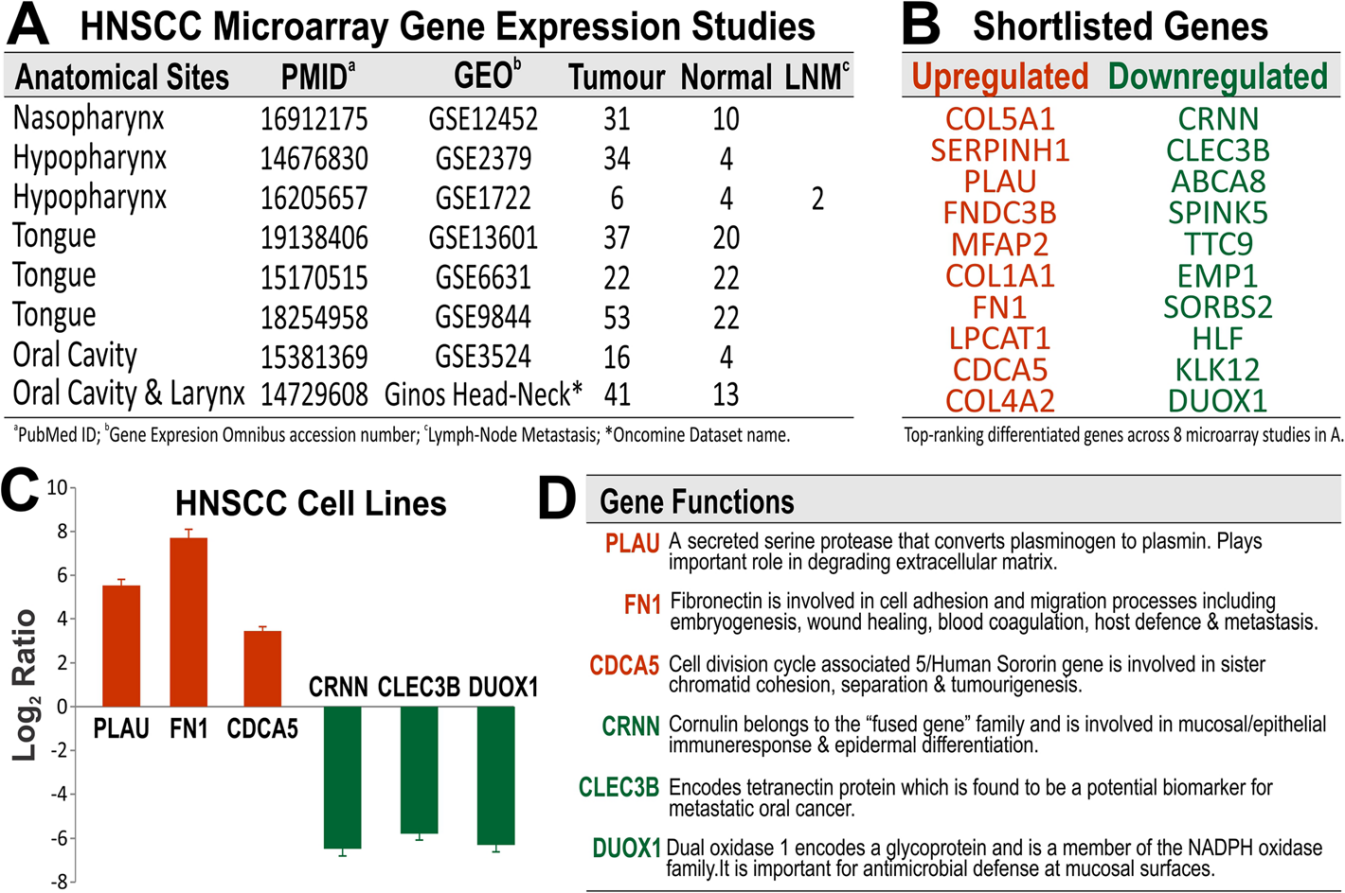
Bioinformatics meta-analysis of 8 independent microarray studies on HNSCC tissues samples compared with normal oral tissues. **a**, Information for the 8 microarray studies: HNSCC anatomical sites, PubMed ID (PMID) referenced to published paper, microarray data archive (GEO or *Oncomine), number of patients’ tumour, normal and lymph-node metastastic (LNM) samples were as indicated. **b**, Based on statistical ranking of the most differentially expressed genes, top 10 upregulated and top 10 downregulated across the 8 studies were shortlisted for further validation on cell lines. **c**, Relative gene expression mRNA levels (Log2 Ratio) were measured using RT-qPCR and compared in a panel of 8 primary normal human oral keratinocytes (OK355, HOKG, OK113, NOK, NOK1, NOK3, NOK16 and NOK376) and 10 HNSCC cell lines (SCC4, SCC9, SCC15, SCC25, SqCC/Y1, UK1, VB6, CaLH2, CaDec12 and 5PT). We identified 3 most significantly upregulated (PLAU, FN1 and CDCA5) and 3 most significantly downregulated (CRNN, CLEC3B and DUOX1) in HNSCC cell lines and their gene putative functions (from NCBI’s Gene database) are listed in D

Transcriptome datasets were queried in the Oncomine (www.oncomine.org) database. The main inclusion criterion was that the studies must involve comparison between HNSCC tumour samples with normal tissues. Studies using HNSCC cell lines were excluded. At the time of analysis, there were 8 studies eligible for analysis (Fig. 1a). Differentially expressed genes were ranked according to their median *P*-values for over-expression and under-expression. Candidate genes were selected based on their top-ranking positions across the 8 studies resulting in a total of 20 upregulated and 20 downregulated genes were shortlisted (Fig. 1b) and were used for subsequent gene expression validation in our HNSCC cell line models using RT-qPCR.

### Reverse transcription quantitative PCR (RT-qPCR)

The RT-qPCR methodology was performed as described previously [15, 16]. Reverse transcription of purified mRNA were converted into cDNA using Transcriptor cDNA Synthesis kit (Roche Diagnostics Ltd., England, UK) and relative gene expression were performed using SYBR Green I Master (Roche) in the LightCycler 480 qPCR system (Roche) based on our published protocols [17–19] which are MIQE compliant [20]. Thermocycling begins with 95 °C for 5 min prior to 45 cycles of amplification at 95 °C for 10s, 60 °C for 6 s, 72 °C for 6 s, 76 °C for 1 s (data acquisition). A ‘touch-down’ annealing temperature intervention (66 °C starting temperature with a step-wise reduction of 0.6 °C/cycle; 8 cycles) was introduced prior to the amplification step to maximise primer specificity. Melting analysis (95 °C for 30s, 65 °C for 30s, 65–99 °C at a ramp rate of 0.11 °C/s with a continuous 5 acquisitions/°C) was performed at the end of qPCR amplification to validate single product amplification in each well. Relative quantification of mRNA transcripts was calculated based on an objective method using the second derivative maximum algorithm [21] (Roche). Sequences of the qPCR primers used in this study are provided in Table 1. All target genes were normalised to two stable reference genes (YAP1 and POLR2A) validated previously [17] to be amongst the most stable reference genes across a wide variety of primary human epithelial cells, dysplastic and squamous carcinoma cell lines, using the GeNorm algorithm [22]. No template controls (NTC) were prepared by omitting cells/tissue sample during RNA purification and eluates were used as NTCs for qPCR assays to monitor contamination.

**Table 1:**
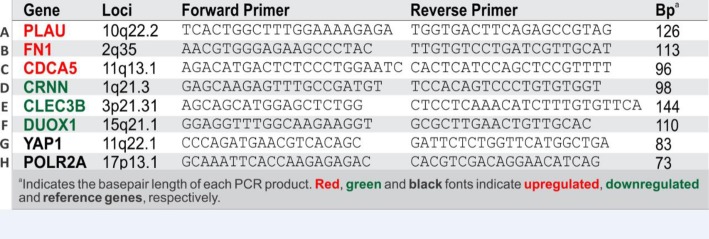
q6 Primer Sequences

### Statistical analysis

Gene expression data were exported from Roche LightCycler LC480 Software as text files for subsequent analysis. Statistical analysis was carried out by the t-test on Graph Pad Prism software and Microsoft Excel and the Mann Whitney U test on SPSS software.

## Results

### Microarray data mining and gene selection

The cancer microarray database Oncomine [23] (www.oncomine.org) was used to select 8 studies which analysed HNSCC cancer samples versus normal as shown in Fig. 1a. From this, top 20 differentially expressed genes were selected based on the reported *P*-value (> 0.001), out of which 10 were significantly upregulated and 10 significantly downregulated (Fig. 1b). Primers for each gene were designed using the Roche Applied Science Universal Probe Library Assay Design Centre for RT-qPCR assays. Initial testing of primer specificity and expression of selected biomarkers was established on cDNA from a panel of 8 normal primary oral keratinocytes and 10 HNSCC cell lines. Based on primer specificity and good reproducibility, three differentially expressed biomarkers were identified, of which, PLAU, FN1 and CDCA5 were found to be upregulated; whereas CRNN, CLEC3B and DUOX1 were downregulated when comparing HNSCC cell lines to normal oral epithelial cells in culture (Fig. 1c). For ease we called them q6 (quantification of selected six biomarkers). The q6 biomarkers were found to be involved in important cellular functions, listed in Fig. 1d.

### Biomarkers identified molecularly distinct HNSCC subgroups

The selected candidate biomarkers were then validated on HNSCC patient tissue specimens by RT-qPCR which provided quantitative data on expression of these selected genes (relative to two reference genes YAP1 and POLR2A) on paired margin and tumour core tissue samples. Individual gene expressions were determined in each margin and core tumour tissue pairs. In order to obtain a clinically meaningful index value from the 6 genes in each patient, we derived an equation to summarise the degree of differential gene expression of the 6 genes between margin and core tissues in each patient:

$$ \mathrm{q}6\ \mathrm{Value}=\left(\mathrm{Sum}\ \mathrm{of}\ \mathrm{Log}2\ \mathrm{Ratios}\ \mathrm{of}\ \mathrm{the}\ 3\ \mathrm{upregulated}\ \mathrm{genes}\right)-\left(\mathrm{Sum}\ \mathrm{of}\ \mathrm{Log}2\ \mathrm{Ratios}\ \mathrm{of}\ \mathrm{the}\ 3\ \mathrm{downregulated}\ \mathrm{genes}\right) $$

**Fig. 2:**
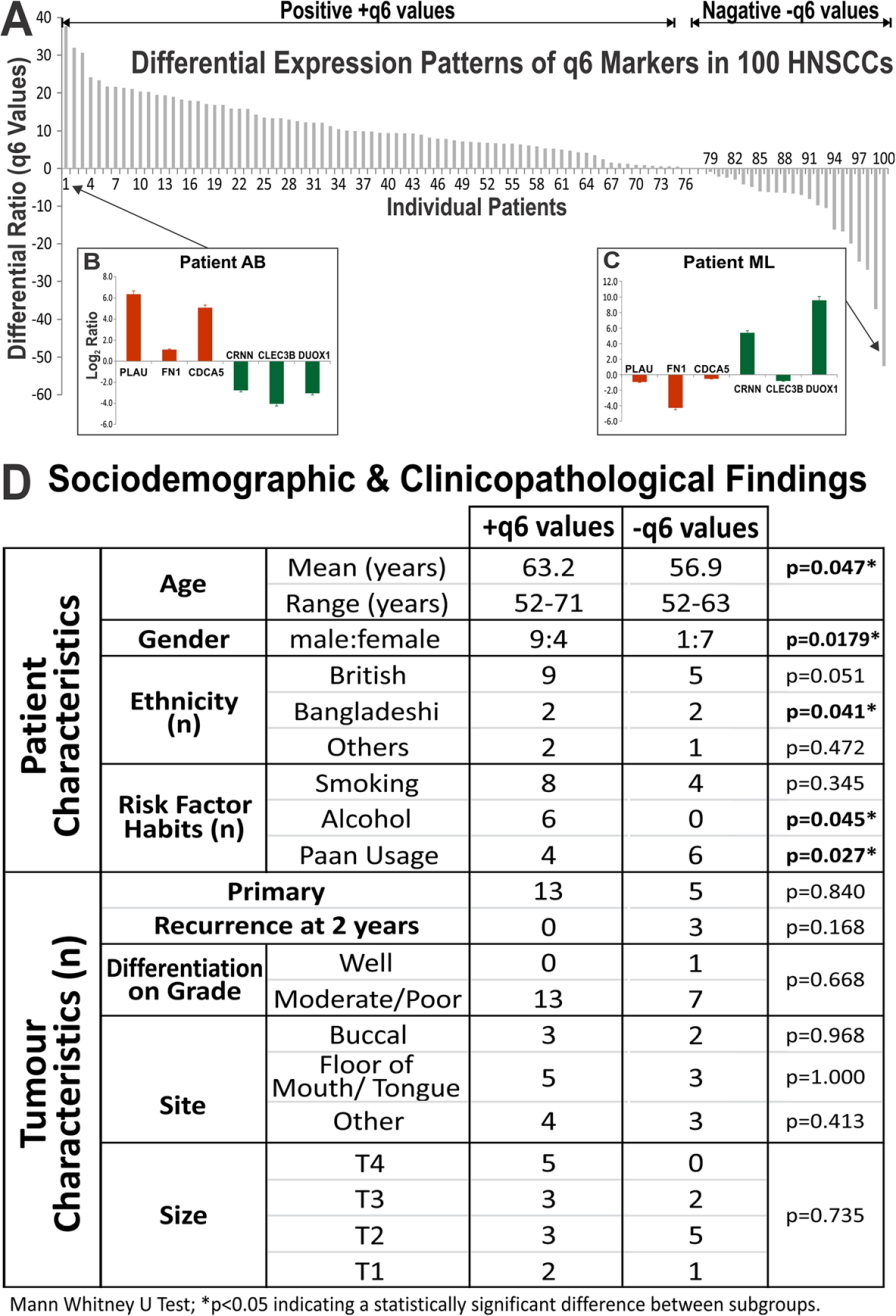
Validation of the 6 markers on 100 HNSCC patients with paired tumour core and margin tissue samples. **a**, The relative mRNA expression levels of each of the 6 markers were measured using RT-qPCR against two reference genes (YAP1 and POLR2A). Differential expression ratios (q6 values) were derived from Log2 Ratio of 3 upregulated markers (PLAU, FN1 and CDCA5) against 3 downregulated markers (CRNN, CLECB and DUOX1). Majority of HNSCC patients showed positive q6 values (indicating the expected expression pattern, as shown in panel **b**) whilst ~ 20% patients showed negative q6 values indicating an inversed expression patterns (as shown in panel **c**). **d**, Statistical analyses of sociodemographic and clinicopathological findings comparing +q6 and -q6 groups

We found that within the 100 patient samples analysed, two molecularly distinct populations could be identified (Fig. 2a). The majority (> 70%) of patients had positive q6 (+q6) values showing the predicted expression of q6 biomarkers from our HNSCC cell line data, with PLAU, FN1 and CDCA5 being upregulated and CRNN, CLEC3B and DUOX1 downregulated. An example of a patient with +q6 expression pattern is shown in Fig. 2b. On the opposite spectrum, about 20% of patients showed negative q6 (−q6) values indicating that these patients showed inverse expression of the q6 biomarkers whereby PLAU, FN1 and CDCA5 were downregulated and CRNN, CLEC3B and DUOX1 upregulated. An example of a patient with -q6 expression pattern is shown in Fig. 2c.

### Clinicopathological analysis of the two HNSCC (+q6 vs -q6) subgroups

In order to understand the different pattern of q6 biomarker expression, patient’s clinical reports were correlated with their molecular findings. Patient and tumour details were collated retrospectively for the 20 most positive and 20 most negative q6 values. In each group, data collection was incomplete for a number of patients as the clinical records were not traceable or the available clinical records were incomplete and therefore these individuals were removed from the analysis. Clinical data from 13 patients with +q6 values was compared to 8 patients with -q6 values. All patients were followed up for at least 2 years following surgical resection of their primary tumours and all tumours were histologically confirmed as HNSCC. The 18 primary HNSCC were excised and subsequently treated with post-operative radiotherapy (RT) with the tissue samples for this study being taken prior to RT whilst the 3 recurrences had originally been resected and all had post-op RT at that time.

We found statistically significant differences in age, sex, ethnicity, alcohol consumption and paan usage among the two groups. In the +q6 group the mean age was 63 with more males than females, while the -q6 group mean age was 56 with more females than males (*P* = 0.04). Additionally, more patients of Bangladeshi descent were found in the -q6 group (*P* = 0.04) (Fig. 2d).

Statistically significant difference was also found in the two groups with regards to associated risk factors. High levels of alcohol consumption in the +q6 group (*P* = 0.04), compared to the -q6 group who were often paan chewers (*P* = 0.02) (Fig. 2d). No difference was found in the smoking habits, tumour site and size among the two groups. Recurrent lesions were only found in the -q6 group although this was not statistically significant (Fig. 2d).

### Prognostic values for q6 biomarkers on other cancer types

**Fig. 3:**
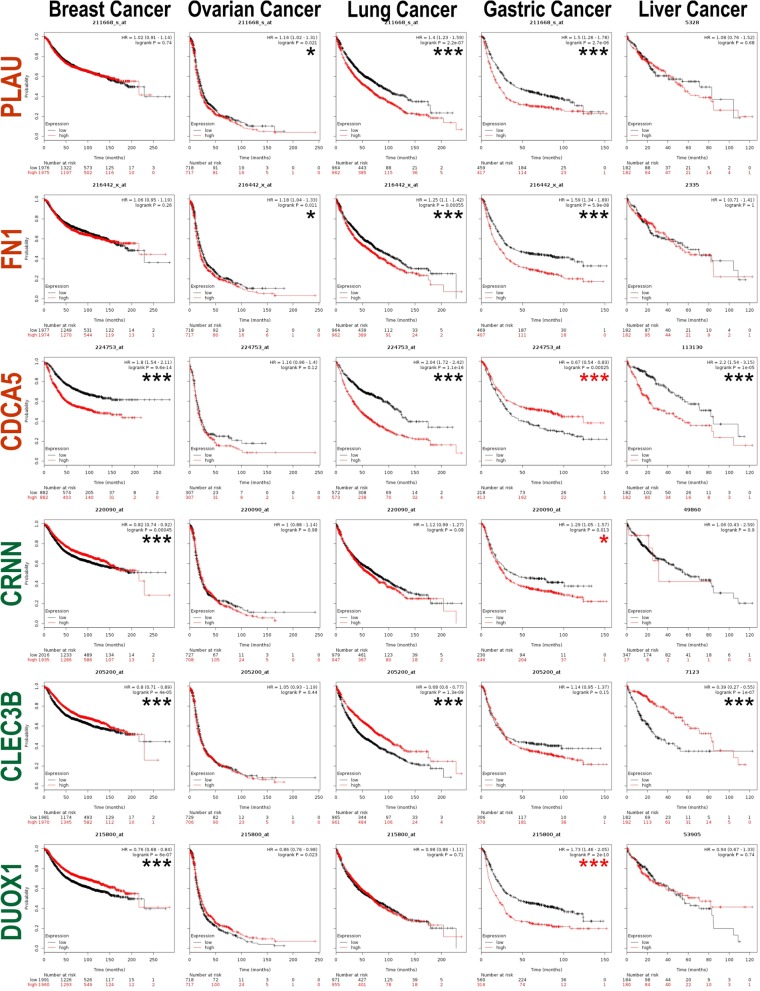
Prognostic significance analysis of the 6 markers on breast, ovarian, lung, gastric and liver cancers using the Kaplan Meier plotter transcriptome database containing 54,675 genes on survival using 10,825 cancer samples (as of 16 Jan 2018). *P < 0.05, **P < 0.01 and ***P < 0.001 showed expected prognostic patterns corresponding to each marker expression levels. Interestingly, * or *** (in red) showed inverse survival curve whereby high expression of e.g., CDCA5 was significantly associated with better prognosis in gastric cancer patients but poorer prognosis in breast, lung and liver cancer patients

We further investigated the prognostic significance of the q6 markers on breast, ovarian, lung, gastric and liver cancers (Fig. 3) using the Kaplan Meier plotter transcriptome database [24] containing 54,675 genes on survival using 10,825 cancer samples (as of 16 Jan 2018). These include 5143 breast, 1816 ovarian, 2437 lung, 1065 gastric and 364 liver cancer patients with a mean follow-up of 69, 40, 49, 33 and 30 months, respectively (kmplot.com). We found that poor prognosis was associated with high expressions of PLAU1 and FN1 on ovarian, lung and gastric cancers. Similarly, high expression of CDCA5 were associated with poor prognosis of breast, lung and liver cancers. Low expression of CRNN, CLEC3B and DUOX1 were associated with poor prognosis of breast cancer. Downregulation of CLEC3B was also associated with poor prognosis in lung and liver cancers. Curiously, gastric cancer showed inverse relationship for CDCA5, CRNN and DUOX1 on prognosis compared to other cancer types (red asterisks; Fig. 3).

## Discussion

In spite of increasing advancements in the management of HNSCC, the long-term survival rate remains unchanged over many decades at about 50%. Currently the mainstay of treatment for amenable cancers is surgical resection and reconstruction followed by adjunctive radiotherapy whilst other tumours can be managed using a combination of chemo and radiotherapy. Unlike breast and lung cancer patients, all HNSCC sufferers are subjected to the same combinations of treatment irrespective of the genetic makeup of their cancer. This is primarily because of the gap in our knowledge regarding molecular biomarkers that can be employed to stratify sub-populations and indicate the most suitable intervention based on the molecular profile of the individual tumour. Some progress towards stratification of HNSCC treatments has been made with the histological identification of HPV-driven HNSCCs as potentially separate clinical entities, but alternative treatment strategies such as deintensification protocols are still in the trial phase (e.g. the ComPARE trial in the UK: www.cancerresearchuk.org/about-cancer/find-a-clinical-trial/a-trial-looking-at-different-treatments-for-people-with-oropharyngeal-cancer-compare). Our data suggests there are multiple other molecularly distinct subtypes of HNSCC, with distinct clinical presentations that may also require stratified treatment approaches.

Through bioinformatics data mining and validation using a panel of 8 normal oral keratinocyte lines and 10 HNSCC cell lines, we identified 6 candidate biomarkers that were differentially expressed, of which 3 (PLAU, FN1, CDCA5) were upregulated and 3 (CRNN, CLEC3B and DUOX1) downregulated in HNSCC. These 6 gene expression profiles in a cohort of 100 HNSCC patients’ tissues, were consistent with the cell line data in the majority of samples (+q6). From clinical correlation this +q6 cohort were found to be predominantly older males who consumed more than the recommended units of alcohol per week which is representative of the majority of HNSCCs found in the UK population as a whole. The alternative genetic profile was an unexpected inverse expression of q6 biomarkers (−q6) observed in about 20% of patients. This study utilised tissue samples collected from an area of East London with the highest concentration of Bangladeshi individuals in the Western world with specific cultural risk factors for HNSCC, such as ‘paan’ (areca nut) usage. This population is known to be at the higher risk of developing HNSCC compared with rest of the UK population and particularly at a younger age and mostly in women due to these cultural influences. Therefore, the finding that patients with -q6 values in this study are markedly different from the positive group in age (significantly younger) and gender (more females) as well as paan users is extremely interesting as it suggests that the q6 response confirms the epidemiological data, indicating that there are two distinct types of HNSCC in our study population. This is further supported by the statistically significant lower reported alcohol consumption in the -q6 group as alcohol usage is low in Muslim cultures while it is a significant issue in the wider UK population.

Of note also is that all recurrent HNSCC were in the -q6 group, although this finding was not statistically significant. This is potentially important as it suggests that the -q6 group could be more prone to recurrence. In addition to the small sample size, it must be noted that to be included in this study the recurrence must have been treated by surgical excision and therefore operable, so this finding must be interpreted with extreme caution but warrants further investigation.

Tissue samples for this study were collected at the time of tumour resection. The decision to treat surgically was made on presenting clinicopathological factors by a multi-disciplinary team of surgeons, oncologists and allied health professionals in conjunction with the patient’s wishes as per NHS best-practice policy. This would suggest the study sample represents a proportion of all head and neck tumours diagnosed in the study period with other tumours being either inoperable (e.g. due to size, position, metastatic spread, patient’s general health or patient wishes) or managed without a tissue sample being generated (e.g. chemo-radiotherapy or laser ablation). Patients in both groups were managed in the same manner, and our genetic analysis indicates that molecularly the tumours in each group were different and showed distinct expression of q6 biomarkers. This suggests that q6 biomarkers can be used to stratify HNSCC patients based on their molecular signatures.

Interpretation of clinical data from a retrospective study of this type must be done with caution particularly when assessing patient treatment modalities, which cannot ethically be influenced by the study design. The various forms of bias and presence of unknown confounders are a significant concern in this study as is the small sample size augmented by the inability to collect patient details on a proportion of each group. Nevertheless, our compelling data indicates the need for a prospective observational study of the correlation between patient factors and HNSCC treatment response.

A further study conducted in our laboratory on a gamma-irradiated resistant oral keratinocyte cell line demonstrated that the downregulation of PLAU, FN1 and CDCA5 appeared to be indicators of the tumour being resistant to radiation therapy (data not shown). Although this finding needs to be verified and validated by further study, the fact that these biomarkers were able to identify tumours that are potentially resistant or responsive to radiotherapy is potentially an important clinical finding as it identifies patients who, for example, may not respond to one treatment modality and therefore would benefit from personalised alternatives.

Further support for the prognostic significance of the q6 markers came from our analyses on breast, ovarian, lung, gastric and liver cancers using the Kaplan Meier plotter transcriptome database [24]. Overall (with exceptions), the 3 upregulated genes (PLAU1, FN1 and CDCA5) were generally associated with poor prognosis in these cancer types when gene expression levels were upregulated. Similarly, the 3 downregulated genes (CRNN, CLEC3B and DUOX1) were generally associated with poor prognosis when downregulated. These further confirms that the 3 upregulated genes tend to be oncogenes whilst the 3 downregulated genes tend to be tumour suppressor genes. Interestingly, gastric cancer showed inverse relationship for CDCA5, CRNN and DUOX1 on prognosis compared to other cancer types investigated. These results indicated that the q6 biomarkers may also have prognostic significance in many other cancer types.

We further looked into the literature to understand the functional significance of q6 biomarkers in HNSCC development and progression. PLAU (plasminogen activator, urokinase) encodes a serine protease involved in degradation of the extracellular matrix facilitating tumour cell migration and proliferation. PLAU has been shown to be a novel biomarker with high tumour expression levels in HNSCC and is linked to decreased survival rate, increased disease progression and relapse [25]. In prostate cancer and laryngeal squamous cell carcinoma, PLAU gene amplification was preferentially found in advanced stage, but not detected in benign lesions, suggesting PLAU may have a tumour stage-dependent expression pattern [26, 27].

FN1 (fibronectin-1) encodes two forms of fibronectin, soluble plasma fibronectin-1 and insoluble cellular fibronectin-1. The insoluble cellular fibronectin is involved in cell adhesion and migration processes including embryogenesis, wound healing, host defence and metastasis. FN1 is also a downstream target of SATB1 oncogene and is up regulated in salivary ductal carcinoma [28], oesophageal squamous cell carcinoma resulting in enhanced cell proliferation and migration [29]. FN1 also induces metalloproteinases, such as MMP9/MMP2 to promote invasion and metastasis [30–32].

The biomarker CDCA5 (cell division cycle associated 5 or human sororin gene) is involved in sister chromatid cohesion, separation and tumourigenesis [33]. A study on lung carcinoma has shown high levels of CDCA5 and its association with poor prognosis [34]. CDCA5 was found to be upregulated in 4 OSCC cell lines and its knockdown led to tumour cell growth inhibition in vitro and in vivo. The same study also found that high levels of CDCA5 immunostaining in OSCC tissues correlated significantly with poorer overall survival [35]. This suggests that CDCA5 has a significant role in OSCC progression, targeting CDCA5 may be a potentially useful diagnostic and therapeutic approach for OSCC patients.

CRNN (cornulin) also known as squamous epithelial heat shock protein 53 belongs to the “fused gene” family and is involved in epithelial immune response and differentiation [36]. In oesophageal squamous cell carcinoma, it is 5-fold downregulated in 89% of cases during transformation from normal to neoplastic cells [37]. Significant loss of CRNN expression is associated with advanced stage, invasiveness, lymph node metastasis and poor survival [37–39]. CRNN expression is reported to be downregulated in HNSCC [40] through loss of heterozygosity and microsatellite instability [41]. These findings highlight the role of CRNN in tumour progression and a possible prognostic marker to predict disease outcome.

CLEC3B (C-type lectin domain family 3, member B) encodes tetranectin protein which is a potential biomarker for metastatic oral cancer. Decreased levels of tetranectin have been assoiciated with cancer progression [42]. In ovarian and breast cancer, decreased serum levels of CLEC3B have been associated with poor treatment response [43, 44]. These findings support that CLEC3B may be used as a biomarker for metastasis.

DUOX1 (dual oxidase 1) encodes a glycoprotein and is a member of the NADPH oxidase family. This protein generates hydrogen peroxide and plays a role in antimicrobial defense at mucosal surfaces. It has been found that in 50% of lung cancers NADPH oxidase DUOX1 and DUOX2 go under epigenetic silencing via hypermethylation of CpG-rich promoter regions. Introducing normal levels of DUOX1 into lung cancer cell lines increased cell migration and wound repair without affecting cell growth [45]. The prognostic value of DUOX1 expression is highlighted in liver cancer with low levels of expression, while normal levels were indicative of disease-free survival [46]. To date the potential use of DUOX1 as a diagnostic or prognostic tool has not been explored in HNSCC.

## Conclusions

We present the first reported correlation of distinct molecular signatures in HNSCC with the clinical presentation of the disease. Larger scale longitudinal studies are now warranted to establish the linkage between these different molecular subtypes and disease progression or treatment response. This is an important step towards the ultimate goal of improving outcomes by utilising personalised molecular-signature-guided treatments for HNSCC patients.

## Data Availability

All microarray datasets used in this study are available in the Gene Expression Omnibus and Oncomine databases. Accession numbers for each dataset have been listed within the manuscript. Any supporting data not included in this manuscript or reagents used in this study, which are not commercially available, will be provided to readers following a written request to the corresponding author.

## Abbreviations

cDNA: complimentary DNA
HNSCC: Head and neck squamous cell carcinoma
HPV: human papilloma virus
LNM: lymph-node metastatic
NTC: no template control
RT: radiotherapy
RT-qPCR: reverse transcription quantitative polymerase chain reaction
